# Phono-spectrographic analysis of heart murmur in children

**DOI:** 10.1186/1471-2431-7-23

**Published:** 2007-06-11

**Authors:** Anna-Leena Noponen, Sakari Lukkarinen, Anna Angerla, Raimo Sepponen

**Affiliations:** 1 Pediatric Cardiology, Jorvi Hospital, Department of Pediatric and Adolescent Medicine, Helsinki University Central Hospital, Helsinki, Finland; 2 Applied Electronics Laboratory, Department of Electrical and Communication Engineering, Helsinki University of Technology, Espoo, Finland

## Abstract

**Background:**

More than 90% of heart murmurs in children are innocent. Frequently the skills of the first examiner are not adequate to differentiate between innocent and pathological murmurs. Our goal was to evaluate the value of a simple and low-cost phonocardiographic recording and analysis system in determining the characteristic features of heart murmurs in children and in distinguishing innocent systolic murmurs from pathological.

**Methods:**

The system consisting of an electronic stethoscope and a multimedia laptop computer was used for the recording, monitoring and analysis of auscultation findings. The recorded sounds were examined graphically and numerically using combined phono-spectrograms. The data consisted of heart sound recordings from 807 pediatric patients, including 88 normal cases without any murmur, 447 innocent murmurs and 272 pathological murmurs. The phono-spectrographic features of heart murmurs were examined visually and numerically. From this database, 50 innocent vibratory murmurs, 25 innocent ejection murmurs and 50 easily confusable, mildly pathological systolic murmurs were selected to test whether quantitative phono-spectrographic analysis could be used as an accurate screening tool for systolic heart murmurs in children.

**Results:**

The phono-spectrograms of the most common innocent and pathological murmurs were presented as examples of the whole data set. Typically, innocent murmurs had lower frequencies (below 200 Hz) and a frequency spectrum with a more harmonic structure than pathological cases. Quantitative analysis revealed no significant differences in the duration of S1 and S2 or loudness of systolic murmurs between the pathological and physiological systolic murmurs. However, the pathological murmurs included both lower and higher frequencies than the physiological ones (p < 0.001 for both low and high frequency limits). If the systolic murmur contained intensive frequency components of over 200 Hz, or its length accounted for over 80 % of the whole systolic duration, it was considered pathological. Using these criteria, 90 % specificity and 91 % sensitivity in screening were achieved.

**Conclusion:**

Phono-spectrographic analysis improves the accuracy of primary heart murmur evaluation and educates inexperienced listener. Using simple quantitative criterias a level of pediatric cardiologist is easily achieved in screening heart murmurs in children.

## Background

Although Dr. Laennec's invention, the stethoscope, has been in clinical use for more than 180 years, and electronic stethoscopes with variable amplification gain have been available for over 80 years, it is still difficult to understand auscultation findings [1-3]. The phonocardiogram, first developed in 1894, visualizes auscultatory signals [2,4,5]. The spectral phonocardiogram has proven to be a reliable tool that gives information of whether or not the murmur is pathological. Based on earlier studies and clinical observations, it has been assumed that pathological murmurs involve sounds of higher frequency [2,5]. Phonocardiography and electronic stethoscopy attempt to improve the diagnostic accuracy of cardiac auscultation. In the most recent studies, digital acoustic analysis has demonstrated the validity of these methods [6-11]. Since the 1980's, phonocardiographic research activity had decreased due to the improvements of echocardiography, which yields more visual information. During the past few years, however, the improvements of personal computers have made it possible to design new low-cost, high-quality phonocardiographic devices [12-17]. Spectral phonocardiography emulates the ear and may be ideal for teaching clinical stethoscopy [4]. The phono-spectrogram combines traditional phonocardiogram with time-frequency distribution presentation of the signal. The spectrogram was introduced for heart sound analysis as early as 1955 by McKusik et al, but was afterwards almost forgotten [4,11].

The first evaluation of children's heart murmur is one of the basic tasks of welfare clinic practitioners and school medical officers. However, based on their frequently limited auscultation experience, they might not be able to recognize the innocence of a heart murmur. Even the auscultation skills of pediatric residents have been found to be suboptimal [18-21]. Several healthy children are referred to pediatric cardiologists or for echocardiography. Parental anxiety may also be a reason to refer the patient for unnecessary examinations, and it is important to recognize an innocent murmur as soon as it is found [22-25]. Auscultation training will naturally improve practitioners' listening skills [26-28].

Previous studies have shown that pediatric cardiologists, based on clinical examination, can differentiate innocent from pathological murmurs with high sensitivity (82...92 %) and specificity (76...99%) [29-33]. Even better results have been attained by using advanced signal processing and pattern recognition tools. Artificial neural network-based screening is reported to have 100% sensitivity and specificity [13,14].

The capability of a doctor or a computerized system to differentiate between pathological and innocent murmurs depends on the quality of the pathological murmurs. It is easy to recognize loud murmurs as pathological by means of clinical or digital analysis [9]. Serious defects are seldom missed, and the challenge is actually to detect mild defects. Small muscular VSD, mild PS or AS causes no hemodynamic harm, but they may require endocarditis prophylaxis. ASD secundum may also be hard to diagnose only by auscultation [34-38].

The purpose of this article was to demonstrate the capabilities of a modern digital system for phonocardiographic recording and analysis and to evaluate its potential for differentiating the characteristic features of heart murmurs in children. Another goal was to investigate how the numerical features measured from a phono-spectrogram can be used to distinguish innocent systolic murmurs from pathological murmurs, which are generally hard to identify in a routine clinical examination. This article additionally concludes more than ten years' experience of studying heart sounds.

## Methods

### Data

Heart sounds were collected at the outpatient pediatric cardiology clinics of the rural hospitals in the vicinity of Helsinki from 1995 to 1999. The Ethics Committees of Jorvi (4/95 number 17) and rural hospitals approved the study. The study series consisted of 807 children, whose ages ranged from newborn to 16 years. The recordings were done at random times, i.e. without pre-set dates. The patient roster was not available in advance and every patient with a positive attitude towards the study were included. From each patient, the auscultation finding at the point of maximal intensity was recorded. The patients were either children with murmurs referred for a cardiologic evaluation for the first time or patients with a known diagnosis. In all cases, the diagnosis was confirmed with echocardiography. The control series consisted of children without heart murmur, including children with paroxysmal supraventricular tachyarrhythmia, spontaneously closing ventricular septal defect or ductus arteriosus. Healthy child volunteers were also recorded and included in the control series. Table 1 summarizes the diagnostic findings.

**Table 1:** Recorded heart sounds

***Diagnosis***	***Number***	***%***
Vibratory murmur	310	38.4
Ejection murmur	107	13.3
Venous hum	26	3.2
Other innocent musical murmurs	4	0.5
Ventricular septal defect (VSD)	87	10.8
Pulmonary stenosis (PS)	47	5.8
Aortic stenosis (AS) or coarctation (CoA)	43	5.3
Mitral valve defect with (MI) or without leakage	29	3.6
Patent ductus arteriosus (PDA)	19	2.4
Atrial septal defect (ASD)	17	2.1
Other	30	3.7
No murmur (control)	88	10.9

**Total**	**807**	**100**

### Equipment

A phonocardiographic system developed in Helsinki University of Technology was used to record heart sounds. The system consisted of an electronic stethoscope and a multimedia laptop computer. A parabolic-shaped cup combined with a high-quality electric microphone and variable-gain battery-powered amplifier in the electronic stethoscope gave a flat frequency response in the whole frequency range of usual heart sounds and murmurs (from 75 to 1500 Hz). The laptop computer had standard multimedia capabilities for audio input and output (16-bit resolution in amplitude, variable recording gain and sampling frequencies from 8 kHz up to 44.1 kHz). Sound signals were recorded digitally using special software for the monitoring and analysis of auscultation. The software was specifically written for this project, and it had several end user (general practitioner) friendly capabilities, such as a database for recordings, basic patient information input dialog, real-time graphical monitoring during recording, selectable and changeable digital filters, tunable settings for the graphical display and analysis, free zooming and replaying of the interesting parts and tools for measuring intensity, duration and frequency range. The software was compatible with standard Microsoft Windows (NT, 2000, XP) environments.

The examiner heard the sounds through earphones and monitored the signals on the computer screen. Thus, the recording was completed in almost the same time as traditional auscultation. Some additional effort was needed to enter the patient information. The new generation commercial electronic stethoscopes were also compatible with this recording program. During the recording it was possible to both to listen to the sound and to follow the phonospectrogram from the display screen, the process was completed often in less than ten minutes.

### Methods

In the post-analysis phase, the recorded sounds were re-played, and the fingerprints of innocent and pathological heart murmurs were simultaneously examined visually by using a phono-spectrogram. First the recorded sounds were digitally filtered using pass-band filtering (75–1500 Hz) (the 3rd order Butterworth type high-pass and low-pass filters). These filter settings were selected by a long term subjective trials where the objective was to trim the display to show the details of the murmurs as an experienced practitioner would understand them. For the traditional waveform display the signal was scaled by the signal's absolute maximum. The resulted waveform showed the relative intensities of the heart sounds as the ear recognizes them and thus absolute intensity scaling is not needed. Similarly in the spectrogram the intensities were scaled by finding the maximum intensity on the time-frequency distribution and calculating relative intensities in the decibel scale. Thus the value of 0 dB corresponded to the maximum intensity and value of -60 dB, which is almost unbearable to listen to. Short time Fast Fourier Transform (STFT) with Hanning windowing, 512 data samples (46 ms time resolution) and total of 1024 FFT points (10.7 Hz frequency resolution) were used for calculating the spectrogram.

The three most common innocent murmurs, i.e. vibratory murmur, pulmonary ejection murmur and venous hum, and the five most common congenital heart defects, i.e. ventricular septal defect (VSD), aortic valve stenosis (AS), pulmonary valve stenosis (PS), patent ductus arteriosus (PDA) and atrial septal defect (ASD), are presented as examples of the whole data set.

To differentiate between innocent and pathological murmurs, 50 vibratory innocent heart murmurs, 25 innocent pulmonary or aortic ejection murmurs and 50 mild pathological systolic murmurs with intensity equal to or less than grade 3/6 were selected from a larger heart sound database for more detailed analysis. 14 of the selected pathological cases were small VSD, 5 hemodynamically loading ASD secundum, 8 PS with a pressure gradient less than 30 mmHg, 8 AS with a pressure gradient less than 30 mmHg, 3 bicuspid aortic valves with velocity less than 2.0 m/s, 1 mild CoA with a measured 15 mmHg RR difference, 1 hypertrophic cardiomyopathy (HOCM) with a septum thickness of 12 mm but with laminar aortic flow, 5 mitral leakage with or without prolapse (MI) and 3 tricuspid valve leakage (TI).

### Parameters

The timings, the intensities and the frequency contents of the sounds were manually extracted from the phono-spectrogram using the software's graphical measurement tools (see any of the Figures from 1, 2, 3, 4, 5, 6, 7, 8). First, the whole recording is listened to carefully. The locations of S1 and S2 are recognized from the graphical presentation showing a moving marker over the phono-spectrogram during replaying. Then, a shorter interesting area containing three whole heart cycles is selected by zooming the signal. The timings are read from the time-scale (at the bottom) by graphically studying both the phonocardiogram and the spectrogram, deciding the duration of the sound events and dragging the marker over the selected time segment. The intensities of S1, S2 and the murmur are read from the scale (on the right) by moving the marker with the mouse over the phonocardiogram. The loudness of the murmur was estimated from the phonocardiogram by comparing the maximum amplitude of the murmur to the average value of the maximum first and second heart sounds Finally, the frequencies in Hertz are read by moving the marker over the spectrogram. The typical intensity for the high and low frequency limits of the systolic murmurs was around -45–50 dB.

**Figure 1:**
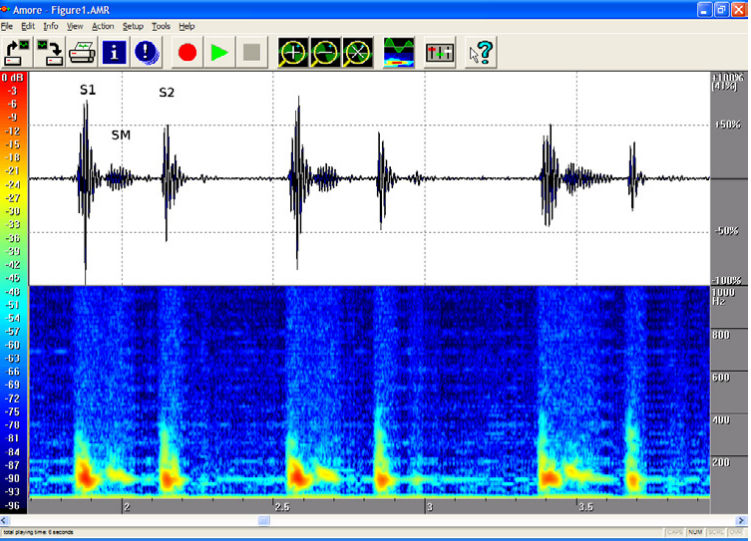
**Innocent vibratory murmur in a 7-year-old**. On auscultation, the typical early and midsystolic low-vibrating murmur is best heard at the left fourth intercostal space and the apex (S1 = First heart sound, S2 = Second heart sound, SM = Systolic Murmur). The phonogram shows a rising and falling early-to-midsystolic murmur, and the spectrogram shows a typical dense configuration and a descending frequency with a maximum gradient of approx. 150 Hz. The auscultation area was the left fourth intercostal space (LIC4). Duration 56%, peak frequency 149 Hz and volume 32%.

**Figure 2:**
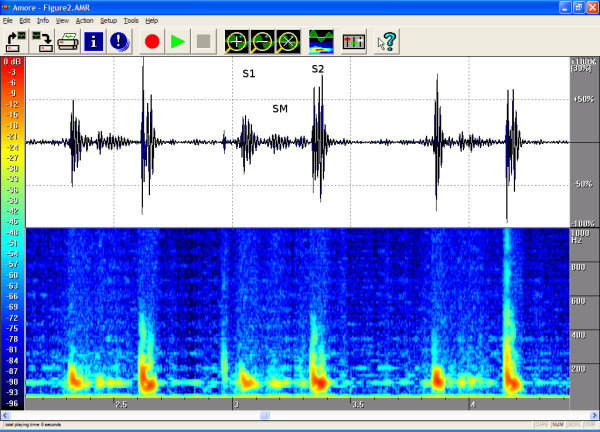
**Innocent pulmonary ejection murmur in a 7-year-old**. This healthy girl with a normal echocardiographic finding had a soft, midsystolic murmur that was second-degree at the most. The spectrogram shows a frequency of about 100 Hz. Auscultation area: left third intercostal space (LIC3). Duration 52%, peak frequency 111 Hz and volume 18%.

**Figure 3:**
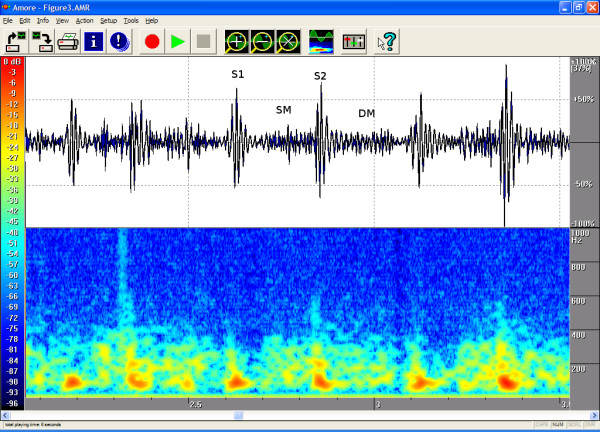
**Venous hum in a 1.7-year-old**. Auscultation revealed a systolic-diastolic second-degree murmur that was loudest in early diastole (DM = Diastolic Murmur). Venous hum is sometimes a misleadingly loud murmur. It is caused by flow in the jugular veins under the clavicle into the superior vena cava. This sound is intensified when the head is turned left and disappears when lying supine. The hum is heard best at the right second intercostal space and medially up behind the sternum. The hum is not necessarily heard on every auscultation. The phonogram shows a rather flat contour, and the spectrogram contains 300 Hz and 400 Hz frequencies at the beginning of diastole, when venous return is fastest. Respiratory sounds are also loud in the area of auscultation, which sometimes makes the interpretation of the curves difficult. Age 1.7 years. Auscultation area: right second intercostal space (RIC2). Duration 100 % of systole and diastole, peak frequency 368 Hz and volume 49 %, at the beginning of diastole.

**Figure 4:**
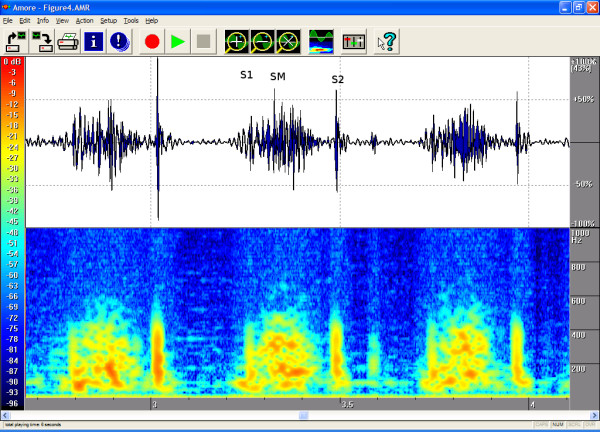
**Aortic stenosis in a 7-month-old**. A third-degree coarse, stenosis-type murmur that does not last until the end of systole is heard in the aortic area. Echocardiography revealed aortic valve stenosis with a 45-mmHg flow gradient. The phonogram shows a diamond-shaped murmur, and the spectrogram contains early systolic sound waves of approx. 500 Hz that coincide with the peak aortic flow. Age 0.6 years. Auscultation area: left third intercostal space (LIC3). Duration 98 %, peak frequency 530 Hz and volume 92 %.

**Figure 5:**
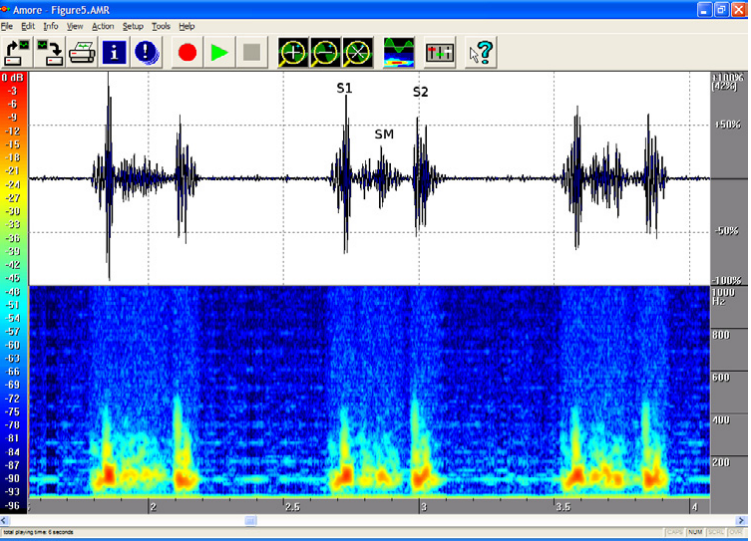
**Pulmonary stenosis in a 9-year-old**. Haemodynamically insignificant pulmonary stenosis, with a 17–22 mmHg flow gradient on echocardiography. On auscultation a second-degree coarse, low-frequency systolic ejection murmur was heard. The phonogram shows the diamond shape compatible with stenotic murmur. Flow velocity is highest at the beginning of systole, and the peak frequencies also occur in early systole, with a descending frequency contour. The findings resemble ASD, the main difference being that P2 is quieter than normal. Because the stenosis is mild, however, P2 can be heard. Area of auscultation: left second ic space (LIC2). Duration 90%, peak frequency 264 Hz, volume 52 %.

**Figure 6:**
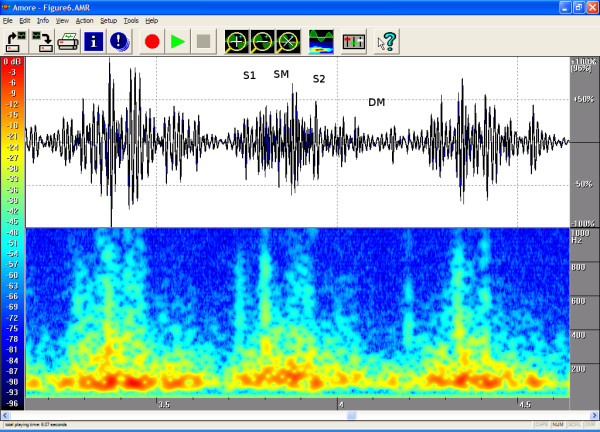
**Ductus arteriosus in a 2.6-year-old**. On auscultation, a fourth-degree continuous murmur with throbbing pulses. Echocardiography showed a large ductus arteriosus but no other defects. The phonogram shows a consistent (systolic-diastolic) murmur that becomes louder towards endsystole and is quieter in diastole. The flow is rapid in systole, which is when the spectrogram shows the highest frequencies. Area of auscultation: left second ic space (LIC2). Pansystolic (duration 100 %) and pandiastolic, peak frequency 786 Hz and volume 150%, both in systole.

**Figure 7:**
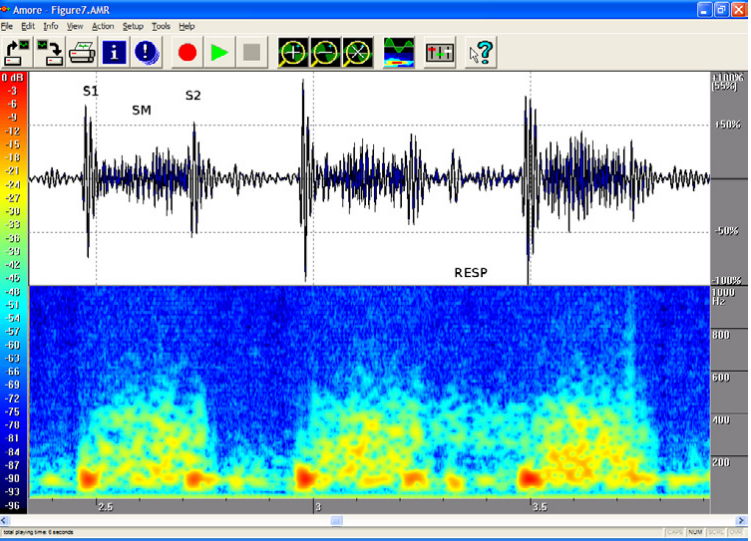
**Fairly large perimembranous VSD in a 7-month-old**. A fairly large perimembranous VSD with normal pulmonary artery pressure (RESP = Respiration). On auscultation a fourth-degree systolic murmur was heard, and on palpation a small thrill was felt in the left third intercostal space. The phonogram shows a pansystolic murmur, but S1 and S2 are distinguishable. P2 is not enhanced and is split normally. This also suggests that pulmonary resistance is normal. Area of auscultation: left third ic space (LIC3). Pansystolic (duration 100 %), peak frequency 523 Hz and volume 61%.

**Figure 8:**
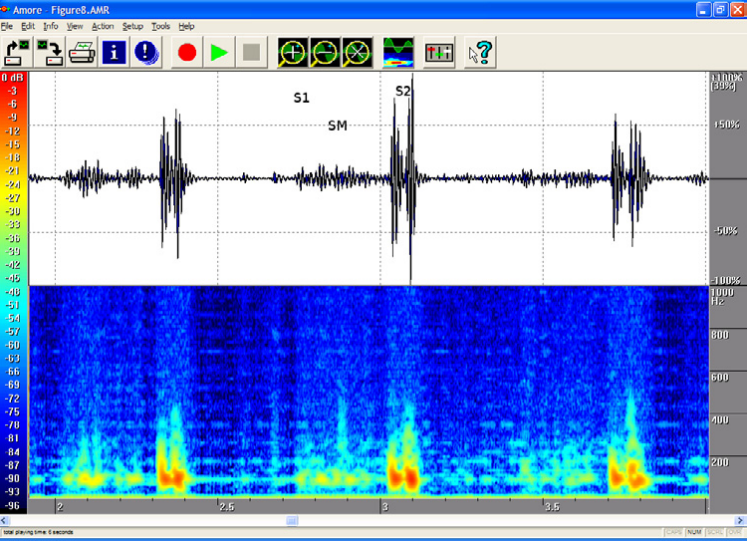
**ASD in an 11-year-old**. On auscultation a quiet systolic ejection murmur was heard in the pulmonary area. It resembled the physiological sound but was longer. The second heart sound was clearly and constantly split. The finding is well depicted on the phono- and spectrograms. Echocardiography showed a 13 x 8 mm secundum type ASD. Pulmonary artery flow velocity was 1.7 m/s and aortic flow 0.9 m/s. Because the ASD secundum murmur is caused by increased pulmonary artery flow, the sound is sometimes difficult to distinguish from the physiological sound. In such cases, attention should be given to the possible splitting of the second heart sound. Area of auscultation: left second ic space (LIC2). Duration 91 %, peak frequency 126 Hz, volume 19 %.

### Statistics

To find statistically significant parameters, two-tailed heteroscedastic t-test for independent samples was used to calculate the p-values. Three sequential beats were manually selected from each recording. The mean value and standard deviation of the parameters over three beats were calculated. Based on the statistical results, the relative duration of the systolic murmur (percentage of murmur duration out of the interval between the end of S1 and the beginning of S2) and the occurrence of intensive high-frequency components were used as criteria for testing the pathology of the murmur.

## Results

### Illustrative examples of most common heart murmurs

The system was able to display and reproduce cardiovascular sound events. The musical murmur caused by harmonic movements of the heart or the vasculature, of which vibratory innocent murmur is a good example, was usually visualized as a well-defined area or line in the spectrogram. Innocent systolic murmur appeared to have a lower peak frequency, below 200 Hz, and shorter duration than pathological murmurs, and it always faded before the second heart sound. The higher the velocity of flow in echogardiography, the more intensive the murmur and the wider the frequency scale. This phenomenon was clearly visible in the spectrogram.

The attached illustrations (Figures from 1, 2, 3, 4, 5, 6, 7, 8) show examples of the most typical murmurs in children. The upper part is a traditional phonocardiogram showing the relative amplitude of the sound. The lower part, i.e. the spectrogram, shows the sound intensity as colors on a frequency scale of 0–1000 Hz. The corresponding intensity scale in decibels is shown on the left. The duration, peak frequency and volume of the murmur were estimated based on a mean of three sequential beats. Volume was compared to the mean of the amplitudes of S1 and S2, obtained from the traditional phonocardiogram.

### Quantitative comparison of innocent and confusing pathological systolic murmurs

The mean and standard deviation of the patients' ages for the three groups are presented in Table 2. The patients with innocent vibratory murmurs were referred for examination at a younger age than those with ejection murmurs, which seemed confusing to the examiners and were referred closer to school age. The pathological murmurs in this series included recordings of both new patients and repeated control recordings of outpatients.

**Table 2:** Mean and standard deviation of patients' ages in the three groups

***Group***	***Age***
Vibratory	4.8 ± 3.3
Ejection	7.3 ± 5.0
Pathological	6.2 ± 4.9

There were no significant differences in the duration of S1 and S2 between the pathological and physiological systolic murmurs (Table 3). Although S2 was constantly split in ASDs, the duration of S2 in these cases did not differ from the average in the series. By contrast, the pathological murmurs, measured in either absolute or relative terms, were longer than the innocent vibratory or ejection systolic murmurs (p < 0.001).

**Table 3:** Duration of S1, S2, systolic murmur and relative duration of systolic murmur

***Group***	***Duration***			
	***S1 (ms)***	***S2 (ms)***	***Murmur (ms)***	***Murmur (%)***

Vibratory	81 ± 15	69 ± 12	100 ± 21	53 ± 12
Ejection	92 ± 16	75 ± 14	84 ± 36	44 ± 17
Pathological	87 ± 19	77 ± 17	147 ± 49	78 ± 21

**p-value**	**0.47**	**0.029**	**< 0.001**	**< 0.001**

Although the pathological systolic murmurs were somewhat louder than the physiological ones, there were no significant differences between the groups (Relative Amplitude in Table 4). This confirmed that the series fulfilled our aim to select easily confusable cases. However, the pathological murmurs included both lower and higher frequencies than physiological ones (p < 0.001 for both low and high frequency limits).

**Table 4:** Relative amplitude and low and high frequency limits of systolic murmur

***Group***	***Amplitude (%)***	***Low freq limit (Hz)***	***High freq limit (Hz)***
Vibratory	23 ± 9	72 ± 15	161 ± 22
Ejection	20 ± 9	60 ± 9	142 ± 51
Pathological	30 ± 20	52 ± 19	299 ± 133

**p-value**	**0.013**	**< 0.001**	**< 0.001**

**Figure 9:**
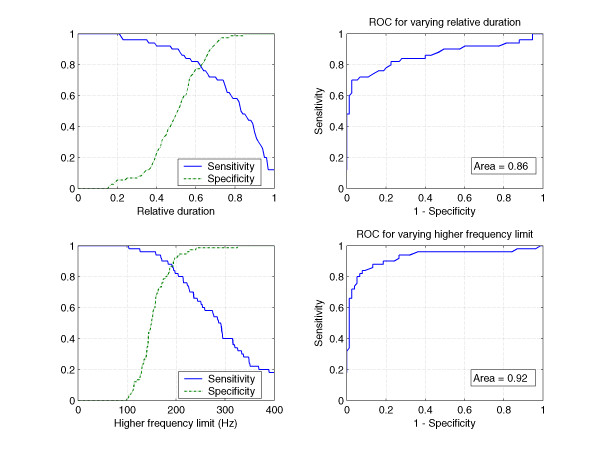
**Single criteria screening effectivity**. (upper left) Sensitivity and specificity for the relative duration of the systolic murmur to differentiate between pathological and physiogical murmurs. (upper right) ROC for the relative duration of the systolic murmur. Area under curve is 0.86. (lower left) Sensitivity and specificity for the high frequency limit to differentiate between pathological and physiological murmurs. (lower right) ROC for the high frequency limit. Area under curve is 0.92.

Sensitivity of 76 % and specificity of 84 % were achieved using a cut-off value of 65 % for the relative duration of systolic murmur to differentiate between pathological and physiological murmurs (Table 5 and Figure 9 – upper left). Using that cut-off, 12 (24%) pathological and 12 (16 %) physiological murmurs were misclassified.

**Table 5:** Capability of relative length to distinguish between murmurs: sensitivity is 76 % and specificity 84 %

	***Pathological***	***Physiological***	***Total***
***> = 65 %***	38	12	24
***< 65 %***	12	63	101

***Total***	50	75	125

When the frequency of 190 Hz for the high frequency limit was used as a classification criterion, both sensitivity and specificity increased to 88% (Table 6 and Figure 9 – lower left). The number of misclassified cases decreased to 6 (12%) in the group of pathological murmurs and to 9 (12%) in the group of physiological murmurs.

**Table 6:** Capability of high frequency limit to distinguish between murmurs: sensitivity is 88 % and specificity 88 %

	***Pathological***	***Physiological***	***Total***
***> = 190 Hz***	44	9	53
***< 190 Hz***	6	66	72

***Total***	50	75	125

**Figure 10:**
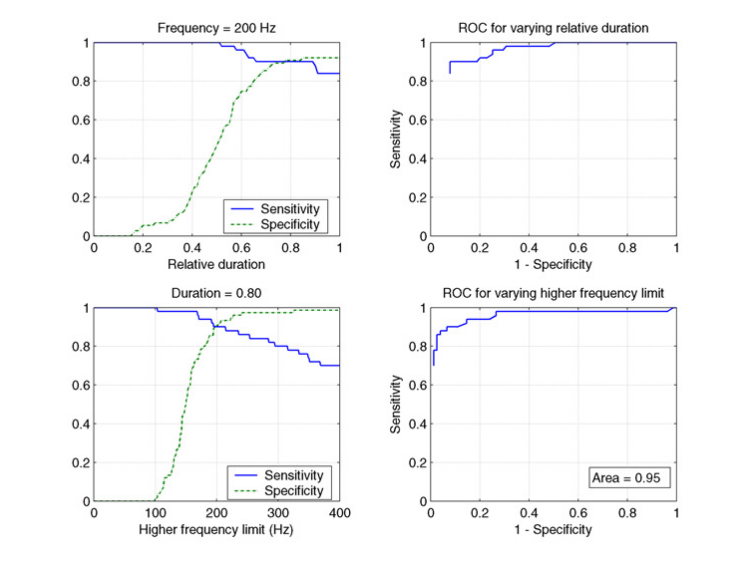
**Sensitivity and specificity for the combined criteria around the optimal point (closest to 100% sensitivity and specificity) to differentiate between pathologial and physiological murmurs**. (upper left) the highest frequency limit is 205 Hz and the relative duration is varied. (upper right) ROC vs. the relative duration around optimal point. (lower left) relative duration is fixed to 0.80 and the high frequency limit is varied. (lower right) ROC vs. the highest frequency limit around optimal point. Area under curve is 0.95.

By combining these two criteria, optimal results were achieved at 80% of the relative duration and with a frequency of 200 Hz as the high frequency limit. Sensitivity approached 90 % and specificity 91 % (Table 7 and Figure 10). There were 5 (10 %) false negative findings and 7 (9 %) false positives.

**Table 7:** Combinated criteria: sensitivity 90 %, specificity 91 %

	***Pathological***	***Physiological***	***Total***
***> = 80 % OR > = 200 Hz***	45	7	52
***All other***	5	68	73

***Total***	50	75	125

## Discussion

Over 90 % of heart murmurs in children are physiological. Moreover, 75 % of them are innocent vibratory murmurs (39, 40, 41, 42). The physician needs to quickly confirm the benign nature of the heart murmur and thus to avoid misdiagnosis [25,43]. The combination of a spectrogram and a traditional phonocardiogram can be an adequate method for distinguishing innocent murmurs from pathological. When quantitative analysis is used systematically, the clinician should know what signals the system is processing and how. Visual analysis is the first step towards understanding automatic analysis, and it is a more reliable way to classify the findings as pathological or non-pathological than auscultation alone. Interpretation of the spectrogram helps to understand the hemodynamic events and the origin of heart sounds. Laminar flow will cause a harmonic wave movement in surrounding tissues, as exemplified by vibratory murmur with a peak frequency of approximately 150 Hz, which has also been reported in earlier studies [39,44,45]. Rapid flow will cause turbulence. The faster the flow velocity, the higher the sound frequencies [4,5]. These findings are also illustrated by the analysis of aortic stenosis or ventricular septal defect [6-10].

It is not simple to estimate absolute volume of heart sound. The point of maximal intensity of the murmur and the thickness of the chest vary and affects on the intensities of the sound components. In our study, the sound volume of murmur was calculated as a percentage of the volume of murmur compared to the mean volumes of S1 and S2. This may be misleading, as we can see in case 4. The volume of the aortic closure sound is decreased because of valvular stenosis, and the volume of murmur is over estimated compared to auscultation findings.

By analyzing the combined phono-spectrogram, it is possible to achieve the level of an experienced pediatric cardiologist in screening heart murmurs. In this study, by using special criteria, sensitivity of 90 % and specificity of 91% were attained. Both sensitivity and specificity were higher than using phonocradiographic or spectral analysis alone. The results were less good than those obtained in previous studies using the methods of advanced signal processing, pattern recognition and artificial networks [10,13-15]. In these previous studies, however, the series consisted of pathological cases with distinct and easily recognizable features of heart sounds and murmurs. We agree that it is easy to distinguish fast turbulent flow with a pressure gradient of over 25 mmHg, and in such cases phono-spectral analysis is reliable. We here examined the grey area, i.e. typical physiological cases compared to mildly pathological murmurs, and the most common pitfalls.

Typically, the screening method is allowed to include some false positive cases, but false negatives are unacceptable. In this study, ten (10) small muscular ventricular septal defects, three (3) cases of mild aortic stenosis, one (1) mild peripheral pulmonary stenosis, one (1) mitral valve prolapse with leakage and one tricuspid leakage fell below the 80 % duration criterion. However, each of these cases exceeded the 200 Hz frequency limit. Three (3) atrial septal defects and one (1) slight pulmonary valve stenosis included frequencies below 200 Hz, but each of them was sustained for over 80% of the systolic duration. Five (5) of the pathological murmurs were not caught with either of these two criteria. One (1) of them was a clinically insignificant tricuspid valve leakage, one (1) a very mild mitral valve leakage, one (1) a bicuspic aortic valve without stenosis and one (1) a bicuspic aortic valve with slight stenosis (pressure gradient 16 mmHg). The last and the worst missed pathological murmur was recorded from a three-year-old boy with hypertrophic cardiomyopathy (thickness of ventricular septum 12 mm). Seven out of 75 innocent murmurs exceeded one of the defined screening criteria. None of the ejection murmurs exceeded the 80 % duration limit, and 3 cases exceeded the frequency limit. All of these cases were aortic ejection murmurs with aortic velocity of 1.5–1.8 m/s. Of the vibratory murmurs, one (1) continued for over 80 % of the systolic duration, three (3) contained frequency components of over 200 Hz, but none of them exceeded both limits.

Slight tricuspid leakage is a harmless finding. Mitral valve leakage and bicuspic aortic valve should be recognized because they require endocarditis prophylaxis. The patient with mitral leakage was a fearful retarded boy, and the recorded signal was weak in amplitude, which is why the highest frequencies were missed in the analysis, but the murmur was sustained until the beginning of the second heart sound, implying a pathological finding. The murmurs due to a bicuspic aortic valve without stenosis or with slight stenosis did not differ from the hyperkinetic aortic ejection murmur. The murmur of the patient with hypertrophic cardiomyopathy resembled vibratory innocent murmur [39]. The difference between these murmurs was hardly seen in the spectrogram. Typically, the frequency of vibratory innocent murmur decreases within the systolic interval, while in this case of hypertropic cardiomyopathy it remained constant. The ECG finding was pathological, and the diagnosis was known beforehand. This particular murmur was rather loud, 31% in spectral analysis and 3/6 estimated clinically. However, 10 (20%) of vibratory murmurs were equal to or louder than 30% measured from phonogram.

As the examples show, the method did not recognize hypertrophic cardiomyopathy or mild aortic valve malformation and failed with the mitral leakage, because the recording was suboptimal. We recommend an echocardiography, when the vibratory or aortic ejection murmur is repeatedly found in adolescent age or when the recording is suboptimal. Naturally, young babies need always an echocardiography, because their pulmonary pressure may still be high and the results of heart sound analysis can be misleading.

Modern electronic stethoscopes are suitable for heart sound recording. Visual presentation of the auscultation finding provides an opportunity to study more objectively and quantitatively the timing, quality (frequency contents) and intensity of different heart sound and murmur components and helps us to understand and learn about the hemodynamics of the heart and the origin of cardiac murmurs.

Computer analysis is a powerful tool in teaching an unexperienced listener the art of auscultation [4,11,46]. Furthermore, we believe that computer analysis can be a promising new diagnostic method that has a potential to reduce medical costs by eliminating unnecessary referrals, visits to specialists and echocardiograms, especially when the systolic murmur is weak. Recording of auscultation findings also makes teleconsultations possible [47-49] and gives an opportunity to save cardiac or respiratory sounds, phonograms and spectrograms in electronic patient documents.

## Conclusion

Phono-spectrographic analysis of heart murmur in children is a useful additional method for the clinical approach and an interesting educational tool as well. The 90 % sensitivity level of the tool may well be at the level of a trained paediatrician. However, it should be considered a single, suboptimal screening tool. As the case of HOCM indicates, ECG and echocardiography are advisable if the intensity of the murmur is fairly high and frequently audible. Patient history and careful physical examination remain the cornerstones in clinical practice.

## Competing interests

The author(s) declare that they have no competing interests.

## Authors' contributions

ALN carried out most of the clinical work, extracted the numerical data for the statistical analysis and wrote the clinical aspects of the manuscript. SL designed the recording system, performed the statistical analysis, wrote the technical description of the system, and reviewed the manuscript. AA participated in the system and study design and data acquisition. RS conceived of the study, and participated in its design and coordination and helped to draft the manuscript. All authors read and approved the final manuscript.

## Pre-publication history

The pre-publication history for this paper can be accessed here:


